# Supplementary material for: Effect of Supplementation of Lambs with Whole Cottonseed: Impact on Serum Biomarkers and Infection by Gastrointestinal Parasites under Field Conditions
Source: Metabolites. 2023 Mar 8;13(3):398. doi: 10.3390/metabo13030398 (PMC10056370; doi:10.3390/metabo13030398)
Supplement: Supplementary file 1 [file metabolites-13-00398-s001.zip › metabolites-2219452-supplementary.pdf]

Supplementary Materials

Table S1. Formulation and chemical composition of the experimental trial diets.

**Diets**	**Whole Cottonseed**	**Control**
**Ingredients (%)**		
Corn ground	17.66	35.07
Soybean meal	4.93	1.15
Mineral[1]	1.83	1.83
Limestone	0.80	0.86
Urea	-	1.26
Monensin[2]	0.03	0.03
Whole cottonseed	14.91	-
Maize silage	29.93	29.91
Aruana guinea grass	29.93	29.91
**Chemical composition**		
Dry matter (%)	52.78	54.60
Net Energy for Maintenance (Mcal/ kg)	2.25	2.23
Net Energy for Gain (Mcal/ kg)	0.87	0.87
Crude Protein (%)	14.21	14.88
Metabolizable Protein (%)	8.50	8.40
Ether Extract (%)	3.96	3.08
Neutral Detergent Fiber (%)	36.19	24.04
Acid Detergent Fiber (%)	21.24	11.69
Calcium (%)	0.70	0.70
Phosphorus (%)	0.40	0.40

[1]Mineral composition (kg of product) 155 g Ca, 65 g P, 110 g Mg, 210 g S, 380 mg Se, 83.500 mg Zn, 26.300 mg Mn, 2500 mg I, 2500 mg Co; (Maximicrominer, Maxi Nutrição Animal).
[2]Rumensin (Elanco Animal Health, Greenfield).

Table S2. Mean (± standard deviation) of average daily weight gain values of crossbred lambs naturally infected with gastrointestinal nematodes and *Eimeria* spp., receiving two different diets (whole cottonseed and control) at seven different samplings (0 – 84 days). Between each sample collection there was an interval of 14 days.

		Time (days)						Effects (*p*-value)		
	**G**	0 - 14	14 - 28	28 - 42	42 - 56	56 - 70	70 - 84	G	Days	G x Days
**ADG (g/day)**	WCS	102.38a (± 87.89)	117.85a (± 72.27)	117.46a (± 52.27)	82.14a (± 94.06)	142.06ab (± 87.54)	200.00b (± 60.31)	0.391	< 0.0001	0.028
	Control	105.55a (± 56.32)	107.14a (± 53.11)	117.46a (± 106.96)	131.34a (± 65.01)	94.04a (± 70.72)	258.33b (± 57.26)			

ADG = Average daily weight gain; G = Groups; WCS = Whole cottonseed. Means followed different lowercase letters in the row differ statistically at each time (days) by Tukey test ($p < 0.05$).

Table S3. Mean (± standard deviation) concentrations of the biochemicals analytes of crossbred lambs naturally infected with gastrointestinal nematodes and *Eimeria* spp., receiving two different diets (whole cottonseed and control) at seven different samplings (0 – 84 days). Between each sample collection there was an interval of 14 days.

	**G**	**Time (days)** 0	14	28	42	56	70	84	**Effects (*p*-value)** G	Days	G x Days
**Albumin (g/dL)**	WCS	2.64A (± 0.47)	2.66A (± 0.46)	2.74A (± 0.42)	2.57A (± 0.37)	2.54A (± 0.48)	2.70A (± 0.43)	2.77A (± 0.35)	0.005	0.116	0.761
	Control	2.96B (± 0.41)	2.86B (± 0.33)	2.86A (± 0.29)	2.71B (± 0.30)	2.63A (± 0.35)	2.77A (± 0.33)	2.81A (± 0.28)			
**Total Protein (g/dL)**	WCS	6.16ab (± 0.94)	5.91ab (± 0.64)	6.04ab (± 0.61)	5.92ab (± 0.58)	5.76a (± 0.85)	6.09ab (± 0.59)	6.27b (± 0.42)	0.785	0.047	1.000
	Control	6.14ab (± 0.62)	5.96ab (± 0.44)	6.12ab (± 0.44)	5.95ab (± 0.59)	5.79a (± 0.71)	6.09ab (± 0.76)	6.24b (± 0.61)			
**Globulins (g/dL)**	WCS	3.51 (± 0.76)	3.26 (± 0.42)	3.30 (± 0.38)	3.34 (± 0.42)	3.22 (± 0.51)	3.39 (± 0.39)	3.49 (± 0.34)	0.059	0.167	0.862
	Control	3.18 (± 0.47)	3.10 (± 0.47)	3.26 (± 0.43)	3.24 (± 0.46)	3.17 (± 0.48)	3.33 (± 0.54)	3.44 (± 0.49)			
**Cholesterol (g/dL)**	WCS	56.08a (± 12.45)	54.67a (± 11.65)	67.22b (± 15.97)	63.90ab (± 12.52)	61.88ab (± 11.94)	61.81ab (± 17.48)	71.79b (± 13.57)	0.263	0.002	0.518
	Control	57.26a (± 15.89)	58.82a (± 10.86)	64.80b (± 10.79)	60.99ab (± 9.13)	57.99ab (± 13.51)	61.40ab (± 11.57)	63.07b (± 14.05)			

G = Groups; WCS = Whole cottonseed. For each variable, means followed by different uppercase letters in the column differ statistically between groups and means followed by different lowercase letters in the row differ statistically at each time (days) by Tukey test ($p < 0.05$).

Table S4. Mean (± standard deviation) concentrations of the biomarkers of oxidative stress of crossbred lambs naturally infected with gastrointestinal nematodes and *Eimeria* spp., receiving two different diets (whole cottonseed and control) at seven different samplings (0 – 84 days). Between each sample collection there was an interval of 14 days.

		Time (days)							Effects (*p*-value)		
	**G**	0	14	28	42	56	70	84	G	Days	G x Days
**TEAC (mmol/L)**	WCS	0.59A (± 0.07)	0.59A (± 0.06)	0.60A (± 0.07)	0.59A (± 0.04)	0.57A (± 0.09)	0.60A (± 0.06)	0.62A (± 0.04)	0.015	0.148	0.974
	Control	0.58A (± 0.05)	0.59A (± 0.03)	0.58B (± 0.04)	0.57B (± 0.04)	0.55B (± 0.06)	0.57B (± 0.06)	0.59B (± 0.06)			
**FRAP (mmol/L)**	WCS	0.43 (± 0.116)	0.44 (± 0.104)	0.47 (± 0.162)	0.44 (± 0.092)	0.47 (± 0.104)	0.46 (± 0.089)	0.50 (± 0.110)	0.191	0.119	0.953
	Control	0.44 (± 0.104)	0.47 (± 0.089)	0.48 (± 0.080)	0.49 (± 0.067)	0.46 (± 0.091)	0.46 (± 0.066)	0.52 (± 0.124)			
**CUPRAC (mmol/L)**	WCS	0.48 (± 0.069)	0.49 (± 0.077)	0.51 (± 0.082)	0.49 (± 0.052)	0.48 (± 0.072)	0.52 (± 0.075)	0.52 (± 0.049)	0.778	0.290	0.507
	Control	0.53 (± 0.050)	0.51 (± 0.043)	0.50 (± 0.053)	0.49 (± 0.038)	0.489 (± 0.063)	0.49 (± 0.045)	0.52 (± 0.056)			
**Thiol (μmol/L)**	WCS	201.54Aa (± 54.16)	199.78Aa (± 48.07)	220.11Aa (± 55.07)	200.37Aa (± 38.54)	202.32Aa (± 46.72)	231.40Aab (± 48.10)	247.39Ab (± 41.79)	0.0009	< 0.0001	0.714
	Control	237.31Ba (± 37.79)	225.86Ba (± 35.63)	219.05Aa (± 32.63)	214.11Aa (± 31.96)	218.83Aa (± 38.13)	247.33Aab (± 40.28)	269.13Bb (± 50.82)			

**Uric acid (mg/dL)**	WCS	0.17 (± 0.038)	0.18 (± 0.055)	0.17 (± 0.039)	0.19 (± 0.054)	0.19 (± 0.066)	0.17 (± 0.037)	0.16 (± 0.054)	0.175	0.439	0.430
	Control	0.17 (± 0.039)	0.16 (± 0.028)	0.18 (± 0.046)	0.19 (± 0.028)	0.19 (± 0.037)	0.19 (± 0.066)	0.17 (± 0.030)			
**PON-1 (IU/L)**	WCS	13.11a (± 3.56)	16.72b (± 2.83)	17.65b (± 2.96)	18.06b (± 3.10)	17.58ab (± 4.01)	14.99ab (± 7.48)	15.65ab (± 6.17)	0.382	0.006	0.561
	Control	13.28a (± 4.38)	17.29b (± 3.93)	16.28b (± 5.26)	16.01b (± 5.16)	15.10ab (± 4.93)	15.86ab (± 5.54)	16.31ab (± 3.58)			
**AOPP (μmol/L)**	WCS	172.69Ab (± 60.56)	155.25Ab (± 47.44)	189.61Ab (± 109.42)	170.53Ab (± 32.96)	156.13Aab (± 37.44)	123.92Aa (± 28.23)	157.86Ab (± 27.05)	0.011	0.0002	0.584
	Control	139.74Bb (± 35.13)	158.02Ab (± 37.01)	154.82Bb (± 38.00)	165.72Ab (± 37.74)	126.91Bab (± 33.74)	109.17Ba (± 29.06)	153.36Ab (± 93.35)			
**TOS (μmol/L)**	WCS	13.88ab (± 6.79)	12.86a (± 5.60)	20.26b (± 19.98)	11.37a (± 3.47)	14.41ab (± 5.67)	11.99a (± 3.51)	15.58b (± 4.80)	0.253	0.031	0.293
	Control	16.59ab (± 5.27)	15.14a (± 5.33)	15.38b (± 5.36)	14.47a (± 5.13)	13.93ab (± 5.68)	13.18a (± 3.99)	20.22b (± 16.94)			
**FOX (μmol/L)**	WCS	51.96A (± 10.35)	52.70A (± 6.82)	57.27A (± 23.18)	52.07A (± 5.35)	52.55A (± 9.12)	49.32A (± 7.22)	56.16A (± 5.56)	0.040	0.078	0.850
	Control	52.37A (± 8.09)	56.20B (± 10.23)	57.36A (± 9.71)	57.79B (± 7.87)	53.14A (± 7.67)	54.45B (± 6.60)	60.68B (± 19.14)			

**d-ROMs (Carr units)**	WCS	122.36Ab (± 31.02)	103.79Aac (± 22.88)	113.88Aab (± 22.81)	93.09Ac (± 19.91)	89.23Ac (± 18.56)	87.06Ac (± 20.93)	101.49Aabc (± 24.83)	0.0008	< 0.0001	0.525
	Control	107.06Bb (± 26.85)	88.80Bac (± 20.55)	94.63Bab (± 17.28)	82.89Bc (± 21.59)	83.18Bc (± 20.93)	86.45Ac (± 21.53)	99.21Aabc (± 27.59)			

G = Groups; WCS = Whole cottonseed; TEAC = Trolox equivalent antioxidant capacity; FRAP = Ferric reducing ability of plasma; CUPRAC = Cupric reducing antioxidant capacity; PON-1 = Paraoxonase-1; AOPP = Advanced oxidation protein products; TOS = Total oxidant status; FOX = Ferric-xylenol orange; d-ROMs = Reactive oxygen metabolites derived compounds means followed by different uppercase letters in the column differ statistically between groups and means followed by different lowercase letters in the row differ statistically at each time (days) by Tukey test ($p < 0.05$).
